# Clinical Features of Cytomegalovirus Retinitis in HIV Infected Patients

**DOI:** 10.3389/fcimb.2020.00136

**Published:** 2020-04-07

**Authors:** Yang Tang, Jianjun Sun, Taiwen He, Yinzhong Shen, Li Liu, Corklin R. Steinhart, Jun Chen, Tangkai Qi, Zhenyan Wang, Wei Song, Renfang Zhang

**Affiliations:** Department of Infection and Immunity, Shanghai Public Health Clinical Center, Fudan University, Shanghai, China

**Keywords:** HIV, CMVR, CMV-DNA, CD4+ T lymphocytes, clinical features

## Abstract

**Objectives:** The purpose of this study was to investigate the clinical features and related laboratory indicators of cytomegalovirus retinitis in HIV infected patients in order to find a suitable laboratory reference guide to aid in the early diagnosis of CMVR, which should improve the prognosis of the severe retinitis.

**Methods:** PLHIVs who were admitted to our hospital from January 2010 to December 2016 were included. The diagnosis of AIDS follows the AIDS Treatment Guidelines. Levels of CMV IgG and IgM were measured by ELISA in order to detect the CMV infection status of the patient. CMV-DNA levels were assessed by a quantitative PCR method, and CD4^+^ T lymphocytes were detected by flow cytometry. Logistical regression was used to analyze the risk factors for CMV retinitis in HIV-infected patients.

**Results:** There were 93 patients with HIV that were also diagnosed with CMV retinitis. After ART, the intraocular pressure, visual acuity, cotton plaque incidence, and CD4^+^ T lymphocyte count were significantly improved, and the yellow-white retinal lesions gradually disappeared. In patients with HIV infections, the CD4+ T lymphocyte count, and peripheral blood quantitative CMV-DNA levels were found to be independent risk factors for CMV retinitis (*P* < 0.05). Patients with HIV infection who had CMV-DNA levels >6,390 copies/mL were associated with more severe ophthalmolgic conditions related to CMV retinitis.

**Conclusion:** Patients with HIV infections with quantitative CMV-DNA levels >6,390 copies/mL have a higher probability of having a diagnosis of CMV retinitis and a worse prognosis than those whose CMV-DNA level is <6,390 copies/mL.

## Introduction

Cytomegalovirus (CMV) infection is a common late stage AIDS complication and may involve a number of organs (Jacobson and Mills, 1988); of these, CMV retinitis is the most common and may lead to blindness if not diagnosed and treated as early as possible. According to one published survey, CMV retinitis of persons infected with HIV were as high as 30% in the pre-ART era (Hoover et al., 1996). Therefore, the early diagnosis of both HIV and CMV infection is particularly important. CD4+ T lymphocyte counts have been used to predict the onset of several ocular infections in HIV-positive persons (Douek et al., 2009); for example, studies have shown that patients with HIV infection with CD4^+^ T lymphocyte counts below 100 cells/μL often have retinal or conjunctival microangiopathy as well as CMV retinitis (Ei et al., 2019). Moreover, 30% of PLHIVs with CD4^+^ T lymphocyte counts below 50 cells/μL have CMV retinitis with blindness mainly due to posterior retinal detachment in these persons (Holbrook et al., 2003).

CMV eye complications in patients with HIV infection often make care of these persons difficult; if they can be diagnosed and treated for CMVR earlier, better outcomes should occur. Interestingly, developing countries such as Ghana often have different types of CMV retinitis manifestations compared to those reported in developed countries (Holbrook et al., 2003). In addition, anterior lesions often cause tumors and external infections while posterior involvement often leads to HIV retinopathy along with opportunistic infections of the retina and choroid (Vrabec, 2004). If not detected and treated in time, posterior complications often lead to severe visual impairment or blindness (Ayena et al., 2010).

For the treatment of patients with HIV infection with ocular complications, it is important to improve their immune status by beginning antiretroviral therapy (ART) as soon as possible (Jabs, 2011). Ganciclovir is currently the more commonly used treatment for CMV retinitis in countries with adequate therapeutic resources (Heiden et al., 2007).

At present, there are few studies focus on the early diagnosis among patients with HIV infection with CMV retinitis. This study retrospectively analyzed the serological and clinical features of CMV retinitis among patients with HIV infection, providing a clinical reference for the early diagnosis and effective management of CMV retinitis.

## Materials and Methods

### Clinical Data Collection

This study enrolled 526 patients with HIV infection who voluntarily underwent ophthalmologic examination and were diagnosed with HIV infection in our hospital from January 2010 to December 2016. The total included 497 men and 29 women with an age range of 26- to 69-years (median: 45.23 ± 11.78). All patients were diagnosed in accordance with the AIDS Treatment Guidelines, and were diagnosed with HIV through molecular diagnostics and met the relevant diagnostic criteria of the AIDS Care Guide (Shen, 2019). Quantitative determination of CMV-DNA levers were performed in blood samples (see below).

### Ophthalmologic Examination and Evaluation

Persons included in the study underwent ophthalmologic examination including visual acuity using a near-sighted visual acuity test, intraocular pressure measurement by a non-contact tonometer, microscopic lesions in the eye using a slit lamp microscope, visual field of the octopus perimetry, and indirect ophthalmoscopy to detect dilated HIV-related retinopathy. When the fundus of patients with HIV infection was found to have lesions, a further TOPCON fundus camera was used for funduscopic photography. For those who had no lesions, the standard fundus color image of the posterior part of the eye was taken. For the peripheral lesions, the posterior pole and the surrounding multi-faceted fundus were photographed. All ophthalmologic examinations and evaluations were performed by the experienced deputy chief physician of the ophthalmology department of our hospital (Ausayakhun et al., 2011; Jirawison et al., 2015). A comprehensive score totaling 100 points from this ophthalmologic examination was compiled; out of 100 points, the higher the score, the more serious the ophthalmologic condition. The patients with HIV infection in our study were divided into two groups and the ophthalmologic score more than 55 named as high score group and other patients with HIV infection were taken as low score group.

### Patient Serum Collection

On the second day after admission, morning fasting venous blood samples were collected from all patients with HIV infection. The method of sample storage and transportation of the samples that were collected above either were used for testing immediately or were stored at 2–8°C for no more than 24 h. If the samples required longer storage the sample was kept at −80°C-degree freezer.

### CD4+ T Lymphocyte Detection

Venous blood was collected from persons of patients with HIV infection, and CD4+ T lymphocyte count assay was performed using a FACS Calibur flow cytometer (BD, USA, model: FACS calibur, USA) and a kit (CD3FITC/CD8PE/CD45PerCP/CD4APC, USA). Automated analysis was performed using the MULTISET software to calculate CD4+ T lymphocyte counts.

### Quantitative CMV-DNA Detection

Human cytomegalovirus DNA in serum was detected using a human cytomegalovirus nucleic acid quantitative detection kit (PCR-fluorescence probe method). The nucleic acids were extracted using AXYGEN-AxyPrep humoral DNA/RNA miniprep kit (AXYGEN, USA). The detection process and results were judged in strict accordance with the kit instructions. Primer 5.0 designed 2 pairs of primers for the immediate early (IE) gene of cytomegalovirus which is conserved region of the virus. The primer sequence was synthesized by Shanghai Shenggong Biotech Co., Ltd. (external primer:IEP4C:5'-TGAGGATAAGCGGGAGATGT-3', IEP4D: 5'-ACTGAGGCAA GTTCTGCAGT; internal primer: IEP4A: 5 '- AGCTGCATGATGTGAGCAAG-3', IEP4B: 5'-GAAGGCTGAGTTCTTGGTAA−3'). The PCR reaction conditions were pre-denaturation at 93°C for 5 min, 91°C for 30 s, 56°C for 1 min, 72°C for 1 min for 30 cycles, and 72°C for 10 min. The PLHIVs' CMV-DNA copy number was quantified using the American ABI Fluorescence Quantitative Detector (ABI, USA, model: ABI-7500).

### Treatment

All study patients with HIV infection underwent routine treatment that included supportive therapy, antiviral therapy, and ART. The patients with HIV infection enrolled in the study were treated with intravenous ganciclovir (5 mg/kg) twice a day and changed to 1 dose/day after 3 weeks of treatment. Diclofenac sodium eye drops and cortisone acetate eye drops can be used for anterior segment inflammation.

### Statistical Analysis

The relationship between CD4+ T lymphocytes, non-CMV retinits and CMV was analyzed using SPSS22.0 software. The data of the normal distribution is expressed as x ± s, and T-test or Wilcoxon Rank test is used for pairwise comparison. The odds ratio (OR) from logistic regression with 95% confidence interval (95% CI) was used to assess the possible risk factors for CMV retinitis. A bilateral *p* < 0.05 indicates a statistically significant difference.

## Results

### Demographic and Basic Clinical Data Analysis of CMV Retinitis and Non-CMV Retinitis in Patients With HIV Infection

Five hundred and twenty six persons underwent funduscopic examination. Among them 93 persons with CMV retinitis (17.68%) were identified. Based on the demographic and clinical data of the CMV retinitis patients and non-CMV retinitis patients, there was no significant difference between them with respect to age (*P* = 0.2712) and gender (*P* = 0.6623). There was, however, a statistically significant difference in CD4^+^ T-lymphocyte count between CMV retinitis and non-CMV retinits patients (*P* = 0.0002). When the CMV-DNA value was <2,000 copies/mL, the CMVR positive rate was 56 (13.5%); when CMV-DNA was more than 2,000 copies/mL, the CMVR positive rate was 37 (33.0%) (see Table 1). There was a highly significant correlation between CMV-DNA copy number and CMV retinitis positive rate (*P* < 0.0001).

**Table 1:** Analysis of demographic and clinical data between CMVR persons of PLHIVs and non-CMVR.

**Characteristics**		**Total number of persons of PLHIVs (*N* = 526)**	**Non-CMVR (*N* = 433)**	**CMVR (*N* = 93)**	***P*-value**
Age	(Median, IQR^*^)	37 (29–48.25)	37 (29.5–50)	35 (28–44.5)	0.1171
	≤30 years old	151 (28.7%)	123 (28.4%)	28 (30.1%)	0.2712
	31–40	174 (33.1%)	138 (31.9%)	36 (38.7%)	
	41–50	83 (15.8%)	68 (15.7%)	15 (16.1%)	
	≥51	118 (22.4%)	104 (24%)	14 (15.1%)	
Gender	Male	487 (92.6%)	402 (92.8%)	85 (91.4%)	0.6623
	Female	39 (7.4%)	31 (7.2%)	8 (8.6%)	
CD4 cell count	(Median, IQR)	38 (14–105)	41 (15–120.5)	26 (6–54)	0.0002
CMV-DNA	<2,000 copies/mL	414 (78.7%)	358 (82.7%)	56 (60.2%)	<0.0001
	≥2,000 copies/mL	112 (21.3%)	75 (17.3%)	37 (39.8%)	

* *IQR, Inter Quartile Range*.

### Improvement of Study Subjects' Condition After Antiviral Therapy

**Figure 1:**
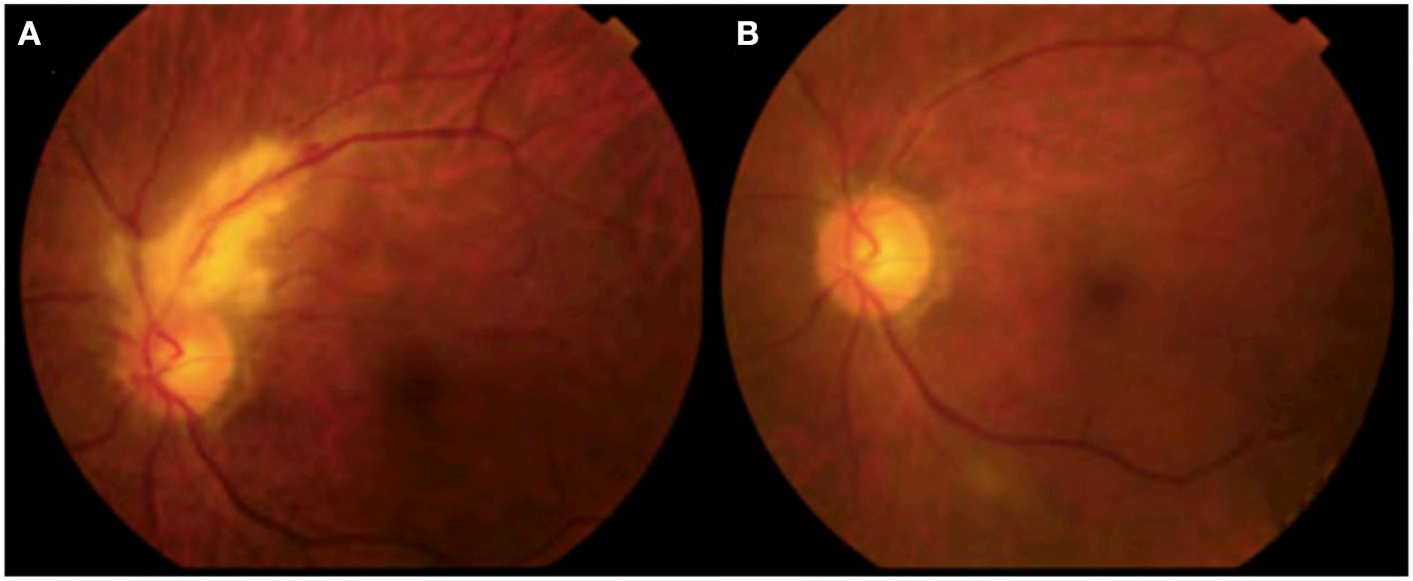
CMVR persons of PLHIVs before and after treatment of ophthalmoscopy. **(A)** Before HAART; **(B)** After 2 weeks of HAART.

The ophthamological status of all CMV retinitis patients improved after antiviral therapy. As can be seen from Table 2, eye pressure, visual acuity, cotton plaque incidence, and CD4^+^ T lymphocyte counts were significantly improved, and CMV-DNA copy number decreased significantly after treatment in this group. White lesions were seen in the vascular arch of the left eyelid before treatment with ART and the area of the yellow-white retinal lesion gradually resolved after 2 weeks of ART in Table 2 and Figure 1.

**Table 2:** Improvement of CMVR patient condition after antiretroviral treatment.

**ART**	**Number of cases**	**Intraocular pressure (mmHg)**	**Vision**	**Cotton plaque incidence (%)**	**CD4 + T lymphocytes**	**CMV-DNA (copies/mL)**
Before treatment	93	13.8 ± 3.0	3.8 ± 0.9	31.2	27 ± 11	3750 ± 1680
After treatment	91	11.2 ± 2.4	4.3 ± 0.8	19.9	88 ± 47	2100 ± 1340
*t*/χ^2^		8.2	6.2	18.2	564.1	3334.5
*P*		0.003	0.021	0.00	0.00	0.00

*ART, Antiretroviral Therapy*.

### Correlation Between CMV-DNA Copies and Ophthalmologic Score

**Figure 2:**
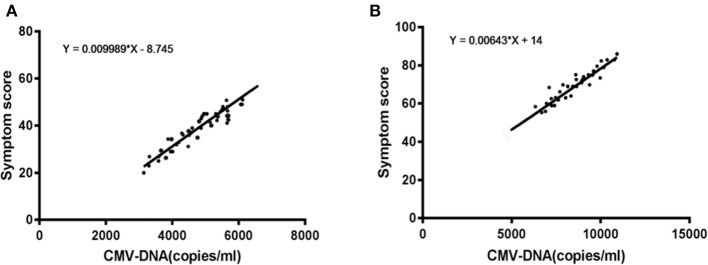
CMV-DNA copies and ophthalmologic score. **(A)** The low score group; **(B)** The high score group.

Those persons who were treated with antiviral therapy for 2 weeks were divided into 2 groups according to the ophthalmology score. Correlation analysis between CMV-DNA copy number and fundus examination score was performed for the two groups (high score group and low score group), as shown in Figure 2 (left panel is the low score group and the right one is the high score group). As the copy number of CMV-DNA increases, the clinical condition of the patient deteriorates. According to the regression equation as described above, the intersection of CMV-DNA copy number of the patients with HIV infection enrolled in the study with different prognoses was obtained. Patients with HIV infection with CMV-DNA > 6,390 copies/mL were much more frequently noted to have severe ophthalmolgic conditions. The sensitivity of this threshold for the diagnosis of severe ophthalmologic conditions in patients with HIV infection was 74.13%, and the specificity was 61.78%, both of which were statistically significant (see Figure 2).

### Logistic Regression Analysis of Factors Affecting CMV Retinitis

Multivariate logistic regression analysis was performed with the confirmed CMV retinitis as the dependent variable and the age, sex, CMV-DNA, CD4+ cell counts as independent variables. The age and gender of patients with HIV infection had no significant effect on the occurrence of CMV retinitis. For patients with HIV infection with CMV-DNA ≥ 2,000 copies/mL, there was an increased risk of CMV retinitis (AOR 2.8, 95% CI 1.7–4.7). In addition, CD4 + cell count <50 cells/μL was found to be another risk factor for CMV retinitis (AOR 1.7, 95% CI 1.01–2.78) (see in Table 3).

**Table 3:** Logistic regression test for the risk factors for CMVR.

**Factors**	**Category**	**Crude odds ratio (COR)**	**95% confidence interval (CI)**	***P*-value**	95% confidence interval (CI)^*^	*P*-value^*^
Age	≤30 years old	Reference	–	–		
	31–40	1.146	0.661–1.987	0.628	0.993 (0.562–1.754)	0.981
	41–50	0.969	0.484–1.939	0.929	0.828 (0.403–1.700)	0.607
	>51	0.591	0.296–1.182	0.137	0.563 (0.276–1.147)	0.114
Gender	Male	Reference	–			
	Female	1.220	0.542–2.748	0.630	1.477 (0.634–3.442)	0.366
CMV-DNA	<2,000 copies/mL	Reference	–	–		
	≥2,000 copies/mL	3.154	1.944–5.118	<0.001	2.847 (1.724–4.699)	<0.001
CD4+ cell counts	CD4^+^ ≥ 50 cell/μL	Reference	–	–		
	CD4^+^ <50 cell/μL	2.098	1.291–3.411	0.998	1.673 (1.007–2.778)	0.047

* *Multivariate logistic regression*.

## Discussion

A previous study reported that the use of PCR for blood CMV-DNA testing is effective in supporting the diagnosis of HIV and CMV co-infection (Jabs et al., 2005). Our study showed that CMV-DNA levels in blood samples from the CMV retinitis group were significantly higher than that in the non-CMV retinitis group. This result indicates that persons with high CMV-DNA load are at high risk of developing CMV retinitis. CMV-DNA has also been reported to be present in aqueous and vitreous samples (Carmichael, 2012). Another study found that CMV-DNA from vitreous and water samples can provide high sensitivity and specificity markers to distinguish between active and inactive CMV infection (Luo et al., 2013). However, the examination of aqueous humor is a traumatic operation with certain risks; indeed, for asymptomatic patients with HIV infection, it is almost impossible to carry out such a procedure on a routine basis. Therefore, when the early diagnosis is needed or when well-experienced staff is not available, serological indicators are still particularly important.

Fifty percentage to 70% of patients with HIV infection with advanced HIV disease have CMV retinitis as manifested by the presence of cotton plaque and retinal hemorrhage (Glasgow, 1997). Pathological changes occurring in CMV retinits are decreased pericytes and swelling of endothelial cells (Mansour et al., 1990). Active CMV retinitis is characterized by retinal necrosis and progressive edema in the white area, and small white satellite lesions in the front of active retinitis (Heiden et al., 2007). These lesions are often classified as painless/granular or fulminant/edema in nature (Liu et al., 2016; Larochelle et al., 2017). In practice, it is often difficult to identify microscopic lesions, which are early manifestations of CMV retinitis in persons with AIDS. PCR detection of autopsy retinas also indicated that CMV-DNA may appear on cotton wool spots (Yoganathan et al., 2016). Treatment of CMV retinitis includes intravenous ganciclovir, oral drug ganciclovir, intravenous sodium foscarnet, intravenous cidofovir, intravitreal ganciclovir or sodium foscarnet and ganciclovir (Jabs et al., 2013). Previous reports have shown that retinopathy progresses over time and occurs in persons who have a long history of advanced HIV infection (Gronborg et al., 2017).

In our study, the mean CD4^+^ count in persons with CMV retinits was significantly lower than that in non-CMV retinitis patients. The prevalence of retinal microangiopathy is known to be inversely proportional to the CD4^+^ count and is common in patients with HIV infection with a CD4^+^ count under 50 cells/μL (Kuppermann et al., 1993). However, it is also common for patients with HIV infection to have a CD4 + count between 200 and 499 cells/μL. Dry keratoconjunctivitis is not associated with CD4^+^ count nor is it associated with HIV severity (Geier et al., 1995).

CMV retinits in persons with advanced HIV disease is usually only diagnosed when the person complains of eye symptoms and ophthalmologic evaluation is then needed to confirm the diagnosis, both of which may be too late to obtain an optimal treatment result. ART in this study improved the ocular symptoms of persons with CMV retinitis. We have demonstrated that the CD4+ T lymphocyte count, and peripheral blood quantitative CMV-DNA levels in patients with HIV infection are independent risk factors for CMV retinitis. We have also shown that persons having a CMV-DNA level of more than 2000 copies/mL and persons with CD4 cell count <50/μl have a higher risk of developing CMV retinitis. However, the finding of person's age is not an independent risk factor for eye complications which is not consistent with research reported from Thailand (Leenasirimakul et al., 2016). In that study, they only recruited patients with HIV infection who had CD4 < 100/μl and there were 16/100 patients with HIV infection diagnosed with CMV retinitis. They found that age (per decade) is one risk factor, albeit weak for the development of CMV retinits (AOR1.70, 95% CI 1.02–2.85). The difference in demographics and age stratification maybe the reason for this difference. Although the subjects in this study were all from China, we feel that there is no reason to believe our results and conclusions do not apply to the diagnosis and treatment of all HIV+ persons with CMV retinitis.

However, despite these important findings, there are some important limitations to this study: the number of cases is relatively small, the study is retrospective, and the study patients with HIV infection are from the same local geographic area and ethnic group. If possible, a larger multi-center clinical epidemiological study is needed with similar laboratory indicators in order to corroborate our finding.

The results of this study can provide serological predictor for the early diagnosis and hence earlier treatment of patients with HIV infection with CMV retinitis. When patients with HIV and CMV infection have a CMV-DNA >6,390 copies/ml, they should be considered at high risk for developing CMV retinitis and therefore the diagnosis can be made early and clinical intervention can be implemented early than the normal standard of care. The prognosis of older patients with HIV infection with antiviral therapy is poor, and it should be closely observed to prevent complications. This study can provide important diagnosis information for improving the early detection rate of CMV retinits patients and hopeful improving their prognosis. When CMV-DNA is >2,000 copies/ml, we must be alert to the possibility of CMV retinitis. If CMV-DNA is >6,390 copies/ml, the possibility of CMV retinitis infection is very high. It is strongly recommended to have ophthalmologic examination. At the same time, the symptoms of patients with HIV infection are often serious.

## Data Availability Statement

The raw data supporting the conclusions of this article will be made available by the authors, without undue reservation, to any qualified researcher.

## Ethics Statement

This research protocols received ethical approval from the Shanghai Public Health Clinical Center Ethics Committee. The committee decided to waive the need for written informed consent from the participants studied in this analysis as the data were analyzed retrospectively and anonymously.

## Author Contributions

RZ, YS, LL, and YT designed the study. JS and YT analyzed the data. TH, JC, TQ, ZW, and WS helped to collect the data. TH and YT provided scientific input. RZ, YT, and CS wrote the paper. All authors read and approved the final manuscript.

### Conflict of Interest

The authors declare that the research was conducted in the absence of any commercial or financial relationships that could be construed as a potential conflict of interest.
